# Life and the British Association

**Published:** 1912-09-14

**Authors:** 


### LIFE AND THE BRITISH ASSOCIATION.

Every year there comes a time when the scientist, be he real or dilettante, has a glorious opportunity of playing to the gallery. The British Association for the Advancement of Science offers him that annual chance, and the temptation is so alluring that few of our scientists have any hesitation in succumbing to it. Some, who do not unduly respect the modern liking for congresses, declare that all these speculations and the dogmatic manner in which they are submitted to the public help no one, and least of all pure science. We cannot go so far as to believe that sweeping assertion; even the silly season has its uses, and if scientists now and again evoke the uproarious merriment of the layman it must, at least, be put to their credit that they have involuntarily and unconsciously added to the gaiety of nations. Usually professional men are so deeply imbued with the belief that their particular science, trade, or profession is sacrosanct that they never dare smile at the silliest conclusions that their major premises, logically followed up, must produce.

Every year the annual meeting of the Association sees the resurrection of an old notion which many of us had deleted from the slate under the impression that decades ago the last word was said for or against it. The beginnings of life is the highfalutin subject with which Professor Schäfer, the president, chose to deal, and his address has been highly applauded and has given much scope to writers in the lay press. It is true there is nothing in it whatever that is new, original, or even interesting. Professor Schäfer simply repeats what his predecessors have said on the subject. He holds that science may yet evolve life. No one blames him for holding that pious hope, but we want from a scientist somewhat more than an expression of opinion. We want reasons, data, and facts, and these Professor Schäfer does not give us. So we have the old controversy over again, which readers of Huxley remember only too well. And the pity of it is that Professor Schäfer cannot claim to have his predecessor's charm and originality. Life, and the possibilities of extending, evolving, and completing it, have indeed overshadowed, so far, most of the more important (because more practical and more scientific) subjects which have interested the Congress. In the Physiological Section Professor Haldane cantered up on his well-known hobby-horse. We thought the animal was ridden to death in class lectures, hospital gazettes, and physiological gatherings. But here it pranced far less exhausted than the immortal Roland, which had to be fed upon wine by the gallant burgesses of Ghent. Dr. Haldane believes in "vitality," which is probably as difficult to define as aquosity, of which Huxley made fun in bygone days. But he went a little off the beaten track of trying a physiological analysis of the soul. Here, again, both in his own remarks and in the debate that followed we have nothing new.

None of our physiologists are philosophers; some are even singularly unenlightened on subjects that trench very closely upon their own speciality. There are some who are sternly materialistic, like Professor Geddes, and others again who are, like Professor Schäfer, singularly hopeful. Probably none is so sanguine as was a greater than all of them, Bertrand, who sincerely anticipated "the final key to the soul now that we know the functions of the liver." Alas! even now, forty years after Bertrand, we do not know the functions of the liver, and we have no more knowledge of the soul than Plato had or Paracelsus. It is permissible for a layman to yawn, therefore, when the Physiological Section of the British Association discusses the soul; in the same circumstances a philosopher will probably go soundly to sleep. The great debate on the origin of life means nothing whatever to those who have a historical knowledge of the matter under consideration, however much it may have amused an unscientific public. Still, as we said at the start, it is the layman and not the scientist who expects epoch-making pronouncements from these annual meetings.

